# Multi Cancer Early Detection by Using Circulating Tumor DNA—The Galleri Test. Reply to Klein et al. The Promise of Multicancer Early Detection. Comment on “Pons-Belda et al. Can Circulating Tumor DNA Support a Successful Screening Test for Early Cancer Detection? The Grail Paradigm. *Diagnostics* 2021, *11*, 2171”

**DOI:** 10.3390/diagnostics12051244

**Published:** 2022-05-17

**Authors:** Oscar D. Pons-Belda, Amaia Fernandez-Uriarte, Eleftherios P. Diamandis

**Affiliations:** 1 Department of Pathology and Laboratory Medicine, Mount Sinai Hospital, Toronto, ON M5G 1X5, Canada; oscardavid.pons@sespa.es (O.D.P.-B.); afernandez@catlab.cat (A.F.-U.); 2 Department of Laboratory Medicine and Pathobiology, University of Toronto, Toronto, ON M5S 1A8, Canada; 3 Department of Clinical Biochemistry, University Health Network, Toronto, ON M5G 2N2, Canada

**Keywords:** multi-cancer early detection, methylation assay, circulating tumor DNA, GRAIL, cancer screening, Galleri test

## Abstract

We recently published some concerns with new technologies which are based on circulating tumor DNA (ctDNA) for early cancer detection. Most of our published criticism, including a commentary in this journal, has focused on tests developed by the biotechnology company GRAIL (their commercial product is also known as The Galleri Test). Scientists from GRAIL provided explanations and rebuttals to our criticism. They also posed some questions. Here, we reiterate our position and provide rebuttals, explanations and answers to these questions. We believe that constructive scientific debates, like this one, can profoundly contribute to advancements in scientific fields such as early cancer detection.

## 1. Introduction

We appreciate the comments of Klein and colleagues [1] related to our previously published commentary [2] regarding multi-cancer early detection by using the GRAIL and related technologies, based on analysis of circulating tumor DNA. Klein et al. raised a number of issues related to our previous contribution and we would like to respond.

## 2. Rebuttals

There is no question that early cancer detection is a key strategy to decrease cancer morbidity and mortality. This effort was initiated about 100 years ago but, unfortunately, with limited success. The limited success is due to the fact that early cancer detection is a complicated issue which includes a number of important components, only one of them being the capability of an analytical test to detect early cancer. For example, early cancer detection must be shown to facilitate stage migration of the cancer and improvement in clinical outcomes, such as overall survival, better quality of life and avoidance of over-diagnosis and over-treatment [3]. Wilson and Jungner’s principles of screening [4] should be a valuable guide to implementing successful cancer screening programs, as we discussed earlier [2]. Klein et al. have some specific questions related to our contribution and we would like to address them one by one.

## 3. GRAIL’s Ongoing Clinical Trials

Klein et al. noted that we did not mention in our previous paper [2] all the clinical trials that are ongoing at GRAIL, which now plan to enroll over 325,000 participants. It is impossible for us to know all planned and ongoing clinical trials of various companies and we congratulate GRAIL for conducting the largest trials that have been undertaken to validate this technology. Many of these trials are due 2 to 10 years from now and it will be interesting to see the results and judge if this technology is clinically useful and to what extent. Independently of theoretical and other limitations that we mention in our previous publications [5,6,7,8,9], these clinical trials will prove the merits and limitations of this technology. Consequently, we will have to wait and see. Some conclusions related to already-completed clinical trials will be mentioned at the end of this commentary.

## 4. GRAIL Test Accuracy

Klein et al. criticize our previous mention of about 80% accuracy with their latest multi-cancer detection test and they elaborate that the actual value for at least two top cancer sites of origin is 88.7%. We consider these differences relatively small and as such they will not change the general performance of the assay or our overall conclusions.

## 5. GRAIL Optimized Assay

Klein et al. mentioned that GRAIL has settled on a commercial test, Galleri, which is based on whole genome methylation analysis, using a targeted assay that covers the most informative regions of the genome for cancer detection and cancer signal origin prediction. Although we mention additional assays that have been tried by GRAIL in the past, we do mention in our publications that one major advantage of the GRAIL technology is that they have settled on a single, well-standardized assay with exceptional specificity [5,6,7,8,9,10]. Consequently, we agree with the authors that the completion of technology development and selection of the most promising assay is a major step forward with this technology.

## 6. Amount of ctDNA in the Circulation

The authors criticize our previous theoretical analyses which are based on the consideration of mutant allele fraction and its correlation to tumor size [7,8]. There is solid evidence suggesting that the mutant allele fraction and the amount of circulating tumor DNA directly correlate with tumor volume [11,12]. However, we agree with the authors that tumor volume and stage are not the only parameters that dictate the amount of circulating tumor DNA. Other factors, including various biological determinants of the tumor, are also major contributors. We also agree that the circulating tumor DNA is highly fragmented, as mentioned in previous contributions [13] and that the GRAIL technology is targeting 30,000 independently informative methylated CpG fragments that cover a significant percentage of the genome. Parenthetically, we ask Klein et al. to elaborate how many of these 30,000 methylation features are critical for cancer detection? We agree with the authors that the estimates of mutant allele fraction, based on tumor size alone is not sufficient for definitive conclusions. This could be a double-edged sword, since patients with approximately the same tumor size may yield highly variable results when ctDNA is used for diagnosis, due to the biology of each tumor and the variable amount of ctDNA in the circulation. Consequently, some patients with about the same tumor size may be highly positive for the test while others will be negative. The authors agree with our previous estimations that there are approx. 3000 haploid genomes in a tube of blood and that a mutant allele fraction of 10^−4^ will mean that there is significantly less than 1 complete haploid genome represented in the circulation. The authors speculate that even with this limitation, their assay could be informative, even if less than 1 genome equivalent is present in the circulation, because they are probing thousands of methylation sites and a whole genome may not be required. However, we have speculated before [7,8], that it is highly likely that the smaller number of ctDNA fragments and the smaller number of methylation sites that are probed will likely weaken the algorithm that is used to detect the presence or absence of cancer, thus increasing the variability in the obtained results (positive or negative results). This issue should be looked at in detail, because it will determine what is the reliability of the assay if the amount of ctDNA is low, and is smaller than the one that was used to develop the prediction algorithm. Because of this limitation, we predicted that many results with the Galleri test will be equivocal or uninterpretable [2,10].

## 7. Tumor Location

We stand by our previous position that in many instances, with this type of testing, there will be situations whereby the test shows the presence of cancer, but the site of cancer development will not be known. This may put surgeons and medical oncologists in a precarious situation because they will not know where to look or operate. The clinical evaluation of the Galleri test will show if this is a major limitation.

## 8. GRAIL’s Published Results

We have indicated earlier that in cancer screening programs, the most important parameter from the patient and physician points of view is the positive predictive value of the test (PPV) [9]. PPV represents the chance of somebody having cancer if the test is positive. The PPV is dependent on three parameters: test sensitivity; test specificity; and disease prevalence within the screened population. The PPV of the Galleri test and other similar tests [10] has not been experimentally confirmed since all published studies are case-control studies, not cohort studies [14]. In case-control studies, the sensitivity and specificity (and the PPV) of screening tests is always over-estimated, because the enrolled patients are already symptomatic (and many of late stage) and the control group may not be diverse enough as the screened population [14].

Nevertheless, we have previously calculated the positive predictive value of the Galleri test for 12 cancer sites, based on the published sensitivity and specificity of the Galleri test [15,16], as well as the prevalence of the disease in the targeted population [5] (Table 1). We found that in almost all of the cancer types, the PPV of this test will be less than 10%, a value that will likely not support an efficient screening program. The latest published data from GRAIL [16] also report rather dismal sensitivities for early cancer (17% for stage I, 40% for stage II and 27.5% for stage I-II) which are likely over-estimations, since these data were derived from case-control studies.

## 9. Conclusions

Despite the expressed optimism, the published data from GRAIL and other similar companies [10] confirm our previous predictions that screening for cancer by using ctDNA will suffer from low sensitivity and low PPV. The speculation that combining these and other ctDNA technologies to make screening viable still requires experimental verification.

## Figures and Tables

**Table 1:** Positive and negative predictive value of a test with 30% sensitivity and 99% specificity for var ious cancers ^1^.

Cancer Type	Cases in US Population	Prevalence Per 100,000	Specificity 99%	Specificity 99%	Sensitivity 30%	Sensitivity 30%	PPV, %	NPV, %
			TN	FP	TP	FN		
Anal	65,000	65	98,935	1000	20	45	2	>98
Bladder	700,000	700	98,300	1000	210	490	17	>98
Colorectal	1,400,000	1400	97,600	1000	420	1360	30	>98
Esophageal	46,000	46	98,954	1000	14	32	1.4	>98
Head and Neck	300,000	300	98,700	1000	90	210	8.2	>98
Liver/Bile duct	83,000	83	98,917	1000	24	59	2.3	>98
Lung	538,000	538	98,462	1000	161	377	13.9	>98
Lymphoma	700,000	700	98,300	1000	210	490	17.3	>98
Ovary	300,000	300	98,700	1000	90	210	8.2	>98
Pancreatic	75,000	75	98,925	1000	23	52	2.2	>98
Myeloma	130,000	130	98,870	1000	39	91	8.3	>98
Stomach	113,000	113	98,887	1000	34	79	3.3	>98
The prevalence of each cancer is shown.								

^1^ The assumption is that the prevalence of these cancers is applicable to US population over the age of 50. Adapted with permission from Ref. [5]. 2020 Oxford University Press.
